# Downmodulation of cholesterol biosynthetic network governs activation of the innate immune response to Japanese encephalitis virus infection

**DOI:** 10.1128/jvi.01972-25

**Published:** 2026-02-04

**Authors:** Sakshi Khera, Kiran Bala Sharma, Yashwant Kumar, Manjula Kalia

**Affiliations:** 1 Regional Centre for Biotechnology, NCR Biotech Science Cluster 682813 , Faridabad, India; 2 Translational Health Science & Technology Institute, NCR Biotech Science Cluster 682813 , Faridabad, India; University of North Carolina at Chapel Hill, Chapel Hill, North Carolina, USA

**Keywords:** Dhcr7, 7-dehydrocholesterol, AY9944, flavivirus, IRF3, IFNβ

## Abstract

Viruses remodel metabolic processes and utilize host lipids for different stages of their life cycle. Our earlier studies have shown that the flavivirus Japanese encephalitis virus (JEV) downmodulates several key proteins involved in sterol and lipid biosynthesis. Through a lipidome analysis, we report that virus infection dysregulates nearly 41% of the cellular lipids in mouse embryonic fibroblasts (MEFs). This manifests as down-modulation of cholesterol, cholesterol esters, and glycerolipids and upregulation of ceramides and several phospholipids. Significant transcriptional downregulation of cholesterol biosynthetic pathway genes was observed in JEV-infected MEFs and mouse bone marrow-derived macrophages (BMDMs). This effect was dependent upon an active interferon (IFN) signaling node. Both knockdown and pharmacological inhibition of 7-dehydrocholesterol reductase (*Dhcr7*), a key regulator of the cholesterol biosynthesis pathway, exerted potent antiviral effects by blocking viral replication and enhancing IFN signaling. Direct supplementation with the *Dhcr7* substrate, 7-dehydrocholesterol (7DHC), showed similar antiviral effects. A partial inhibition of virus replication was also observed in cells deficient for IFN signaling, highlighting an IFN-independent antiviral role of *Dhcr7* inhibition. Overall, these findings underscore the potential of cholesterol biosynthetic pathway inhibition as an antiviral strategy for JEV.

**IMPORTANCE:**

JEV, a mosquito-borne virus, is a leading global cause of virus-induced encephalitis with a significant disease burden in Southeast Asia. In this study, we observe that the cellular lipid composition changes in virus-infected cells, with lower levels of cholesterol and cholesterol esters. We also observe that specific genes of the cholesterol biosynthesis pathway are decreased, and this depends on the activation of the antiviral interferon (IFN) response. We have characterized one specific downregulated gene Dhcr7, which catalyzes the conversion of 7-dehydrocholesterol (7DHC) to cholesterol. Depletion of the *Dhcr7*-specific mRNA, inhibition through drugs, or adding the substrate 7DHC further enhanced the IFN response and blocked virus replication. Our study highlights that downregulation of the cholesterol biosynthetic pathway has the potential to be developed into an antiviral strategy. ** **

## INTRODUCTION

At the cellular level, a productive virus infection involves significant alteration of the host’s proteome and lipidome, which pans out as an intricate interplay between virus replication, innate immune activation, and metabolic rewiring. The lipid composition of several membranes and specific organelles influences every aspect of the virus life cycle, starting from virus attachment and entry to replication and egress (1–5).

Studies have shown that flaviviruses such as West Nile virus (WNV) and Dengue virus (DENV) extensively remodel the cellular lipidome to favor effective virus replication. Mass spectrometry analysis of DENV-infected mosquito cells revealed that 15% of detected lipids differed significantly from those in uninfected cells (6). Similarly, WNV-infected cells showed elevated glycerolipid and sphingolipid levels, which favored virus replication (5). Hepatitis C virus (HCV) infection is also well-characterized in terms of alterations in phospholipid and sphingolipid levels (7, 8).

WNV and DENV also dysregulate cholesterol synthesis and uptake, and inhibiting these pathways has antiviral effects (9–12). Studies have shown that increased expression of cholesterol biosynthesis genes, such as hydroxy-methylglutaryl-CoA reductase (*HMGCR*) and mevalonate diphospho decarboxylase (*MVD*), enhances the replication of DENV and WNV (10–12).

Alterations in the cholesterol metabolic pathway are strongly associated with type I interferon (IFN) signaling (13–17). Selectively reducing the synthesized cholesterol pool has been reported to spontaneously activate type I IFN signaling, priming cells for an antiviral immune response (13). Inhibiting cholesterol via statins can enhance the retinoic acid-inducible gene I (RIG-I)-mediated antiviral response against Sendai virus (SeV) (18). Additionally, limiting cholesterol biosynthesis also increased the production of non-canonical type I interferons, such as IFN-ω and IFN-α16 (18).

Here, we have characterized the interplay between the cholesterol metabolic network and innate immune responses in Japanese encephalitis virus (JEV)-infected cells. JEV is a neurotropic flavivirus and a leading cause of virus-induced encephalitis in Southeast Asia (19). Earlier studies from our laboratory have shown that JEV infection of mouse embryonic fibroblasts (MEFs) resulted in significant downregulation of proteins involved in sterol and lipid biosynthesis (20). In this study, we have performed a lipidome analysis of JEV-infected MEFs. We report that the virus dysregulates nearly 41% of the cellular lipids. This manifests as downmodulation of cholesterol, cholesterol esters, and glycerolipids, and upregulation of ceramides and several phospholipids. We also observe an IFN-dependent transcriptional downregulation of the cholesterol metabolic pathway genes in MEFs and bone marrow-derived macrophages (BMDMs). Knockdown or pharmacological inhibition of a key regulator of the pathway, 7-dehydrocholesterol reductase (*Dhcr7*), exerted a potent antiviral effect through the activation of interferon regulatory factor 3 (IRF3) and type I IFN. Supplementation with the metabolite 7-dehydrocholesterol (7DHC) showed a similar reduction in virus replication and titers, along with enhanced expression of pIRF3. The observed inhibition of virus replication persisted, albeit to a smaller extent in cells deficient for IFN signaling, suggesting that cholesterol biosynthetic pathway inhibition also exerts IFN-independent antiviral effects.

## RESULTS

### Lipidomic profile of JEV-infected MEFs

**Fig 1:**
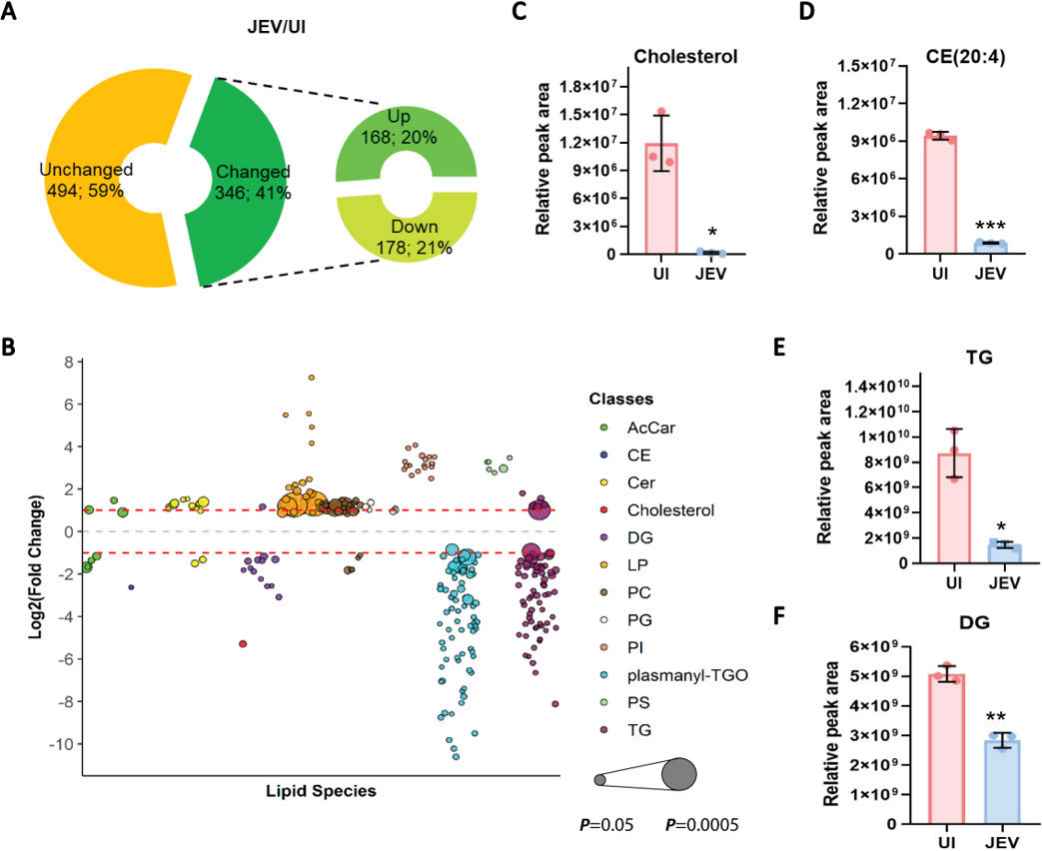
Lipidomic profile of JEV-infected MEFs. (**A**) Pie chart showing the percentage of cellular lipidome altered upon JEV infection compared to uninfected cells. The abundance of lipid species normalized to uninfected control in UI- and JEV-infected conditions was compared to calculate FC (JEV/UI). The thresholds of ≥ −1 and ≥ 1 of log 2 (FC) and *P*<0.05 were used to find the differentially expressed lipid species. (**B**) Bubble plot showing the log_2_(FC) in the abundance of dysregulated lipid species in JEV-infected conditions relative to uninfected cells. Lipid species are grouped into their respective classes and marked with representative colors. Bubble size represents the *P* value calculated from a one-way analysis of variance (ANOVA) *t*-test. (**C–F**) Bar graph displaying the relative peak area that correlates with the lipid abundance normalized to uninfected control of cholesterol (**C**) and CE (20:4) (**D**), TG (**E**), and DG (**F**) in UI- and JEV-infected conditions. Values are represented as mean ± SD. ANOVA with Tukey’s Post hoc test, followed by 2k factor, was used to calculate *P* values. (*P < 0.05, **P < 0.01; ***P < 0.001).

To characterize changes in the cellular lipidome upon JEV infection, we performed a whole-cell-based lipidomic analysis of UI/JEV-infected (multiplicity of infection [MOI] of 2, 24 h) MEFs. Lipids extracted from biological triplicates of UI- and JEV-infected cells were examined using liquid chromatography-mass spectrometry (LCMS)-targeted lipidomic analysis (Fig. S1). A total of 840 lipid species were identified, mainly belonging to carnitines, sterols, sphingolipids, phospholipids, glycerolipids, ceramides, sphingomyelins, and plasmalogens (Data Set S1). The abundance of lipid species in both data sets was compared for further analysis. Principal component analysis (PCA) of UI- and JEV-infected samples confirmed the reproducibility of triplicate samples of each condition and showed that JEV infection is responsible for most of the changes in cellular lipid profile (Fig. S2A). Using volcano plot analysis, we found significantly increased (FC ≥ 2) and decreased (FC ≤ 0.5) lipid species in JEV-infected MEFs (Fig. S2B; Data Set S1). Out of the total, 346 lipid species (41%) showed significant change in abundance (*P* < 0.05, *t*-test), and among them, 168 lipid species (20%) were upregulated, and 178 (21%) were downregulated (Fig. 1A). We grouped the lipid species into their respective lipid classes/subclasses. Major changes were observed in sterols, sphingolipids, phospholipids, glycerolipids, and plasmalogens (Fig. 1B). We found that sterols (cholesterol and cholesterol esters [CE]), glycerolipids (triglyceride [TG] and diglyceride [DG]), and plasmanyl-triacylglycerol (plasmanyl-TG) were downregulated upon infection (Data Set S1). The comparison of the relative peak area, which correlates with the normalized lipid abundance among UI- and JEV-infected conditions, further confirmed the significant reduction in the cholesterol (Fig. 1C), CE (20:4) (Fig. 1D), TG (Fig. 1E), and DG (Fig. 1F) levels. Heatmap analysis of the abundance of dysregulated TG- and DG-specific lipid species in UI- and JEV-infected samples also confirmed the significant suppression of a diverse range of DGs (Fig. S3A) and TGs (Fig. S3B) upon JEV infection.

Several ceramides, phospholipids (Lyso/phosphatidylcholine [LPC/PC], phosphatidylserine [PS], phosphatidylinositol [PI], and phosphatidylglycerol [PG]), and plasmalogens (except plasmanyl-TG) were found to be significantly upregulated in JEV-infected MEFs (Fig. S4; Data Set S1). Using heat map analysis, we compared the abundances of dysregulated species of ceramide (Fig. S4A), PC (Fig. S4B), PI (Fig. S4C), PG (Fig. S4D), and PS (Fig. S4E) among the UI- and JEV-infected data sets and found enrichment in a broad range of phospholipid content upon JEV infection. Most of the enhanced species in PC, PI, PG, and PS classes were enriched with unsaturated fatty acyl chains, suggesting the enhancement of unsaturated degree of lipid content during JEV infection. Collectively, the lipidomic analysis reveals that JEV significantly alters the fibroblast lipid profile and majorly disturbs the subclasses of sterol, glycerolipids, and phospholipids.

### Cholesterol biosynthetic pathway is transcriptionally downmodulated upon JEV infection and interferon signaling

**Fig 2:**
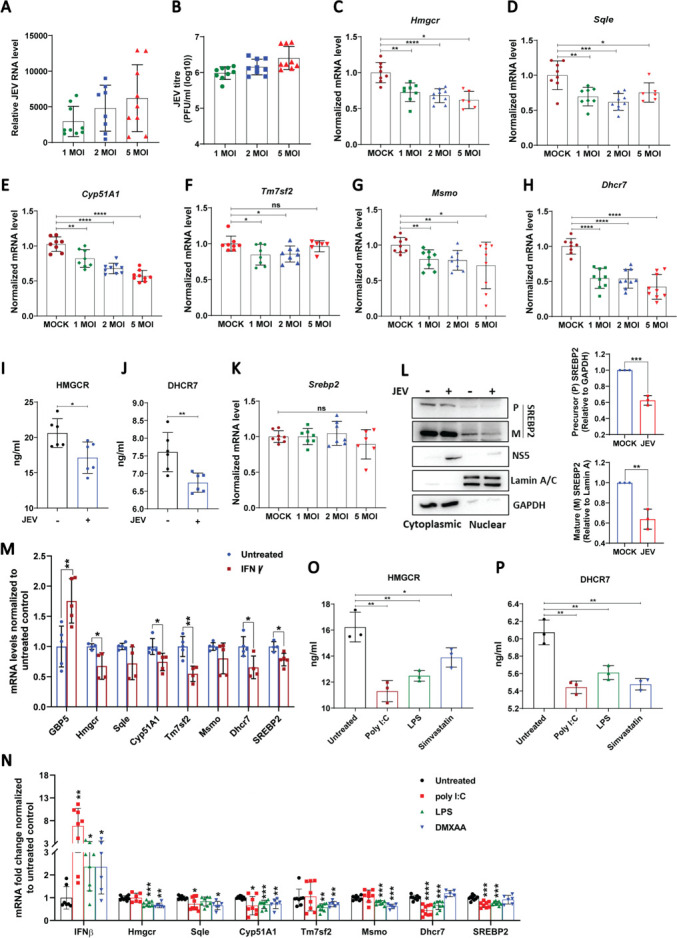
Cholesterol biosynthetic pathway is transcriptionally downmodulated upon JEV infection and IFN signaling. MEFs were MOCK/JEV-infected at MOIs of 1, 2, and 5. At 24 hpi, the RNA levels of JEV (**A**) and cholesterol biosynthetic genes, *Hmgcr* (**C**), *Sqle* (**D**), *Cyp51A1* (**E**), *Tm7sf2* (**F**), *Msmo* (**G**), *Dhcr7* (**H**), and *Srebp2* (**K**) were measured using qRT-PCR, normalized to mock-infected cells. Extracellular viral titers were quantified using plaque assays (**B**). Mock or JEV-infected MEFs were processed for cell lysate preparation at 24 hpi. Protein concentrations in the lysates were determined using bicinchoninic acid (BCA). Equal amounts of protein (30 µg) were loaded onto pre-coated HMGCR and DHCR7 ELISA plates to measure HMGCR (**I**) and DHCR7 (**J**) levels, normalized to those in mock-infected cells. (**L**) Western blot showing cytoplasmic and nuclear fractionation of Mock and JEV (MOI of 2) infected MEFs, blotted with SREBP2, *P*: Precursor form; M: Mature form, Lamin A/C (nuclear control), and GAPDH antibodies. Bar graphs on the right show levels of SREBP2 (*P* relative to GAPDH, M relative to Lamin A) in JEV-infected cells normalized to the respective levels in mock-infected cells. (**M and N**) MEFs were treated with IFNγ (30 ng/mL) for 12 h, poly I:C transfection (1 µg) for 6 h, LPS (1 µg) for 12 h, or DMXAA (100 µg) for 2 h. The mRNA levels of cholesterol biosynthetic genes were analyzed via qRT-PCR, and relative gene expression levels normalized to untreated controls were plotted. (**O and P**) Cell lysates from poly I:C transfected, LPS, and Simvastatin (10 µM)-treated cells were subjected to Hmgcr (**O**) and Dhcr7 (**P**) ELISA, and the results were plotted after normalization to untreated controls. The data presented is mean ± SD of values obtained from three independent experiments. A Student’s *t*-test was used to calculate *P* values (*P < 0.05, **P < 0.01, ***P < 0.001, ****P < 0.0001).

We have previously shown that JEV infection downregulates several proteins that have essential roles in lipid and cholesterol biosynthetic pathways (20). Since we also observed reduced cholesterol levels in virus-infected cells, we analyzed the expression of a few key genes of the cholesterol biosynthetic pathway. JEV-infected MEFs showed MOI-dependent enhancement of viral RNA levels (Fig. 2A) and titers (Fig. 2B), along with significant downregulation of *Hmgcr*, squalene epoxidase (*Sqle*), cytochrome P450 (*Cyp51A1*), transmembrane 7 superfamily member 2 (*Tm7sf2*), methylsterol monooxygenase (Msmo), and *Dhcr7*, with *Dhcr7* showing the greatest reduction ~ 50% (Fig. 2C through H). Protein levels of HMGCR and DHCR7 as determined by high-sensitivity enzyme-linked immunosorbent assay (ELISA) were also significantly reduced in infected cells (Fig. 2I and J). Transcript levels of the major transcription factor of the pathway, the Sterol regulatory element binding protein 2 (*Srebp2*) (21), remained unchanged (Fig. 2K). However, precursor and mature SREBP2 protein levels were found to be significantly reduced in the cytosolic and nuclear fraction, respectively, of JEV-infected cells (Fig. 2L), suggesting downmodulation of the pathway. A similar experiment was performed in BMDMs, wherein JEV infection or IFN-γ stimulation resulted in downmodulation of the cholesterol biosynthetic pathway genes (Fig. S5A through J). Collectively, our data show that JEV infection results in a coordinated downregulation of the sterol biosynthetic pathway and a decrease in cholesterol levels in infected cells. Similarly, DENV-2-infected MEFs (Fig. S6A and B) also showed downmodulation of *Hmgcr* and *Dhcr7* (Fig. S6C and D). Treatment of MEFs with IFN-γ, or stimulation with poly I:C, LPS, STING agonist DMXAA, also showed transcriptional downregulation of sterol biosynthesis (Fig. 2M and N), and a concomitant decrease in protein levels of HMGCR and DHCR7 (Fig. 2O and P). This suggests that the observed downregulation of the pathway is likely to be a broader mechanism linked to innate immune sensing of RNA virus infection.

### Transcriptional downregulation of the cholesterol biosynthetic pathway is dependent upon IFN signaling

**Fig 3:**
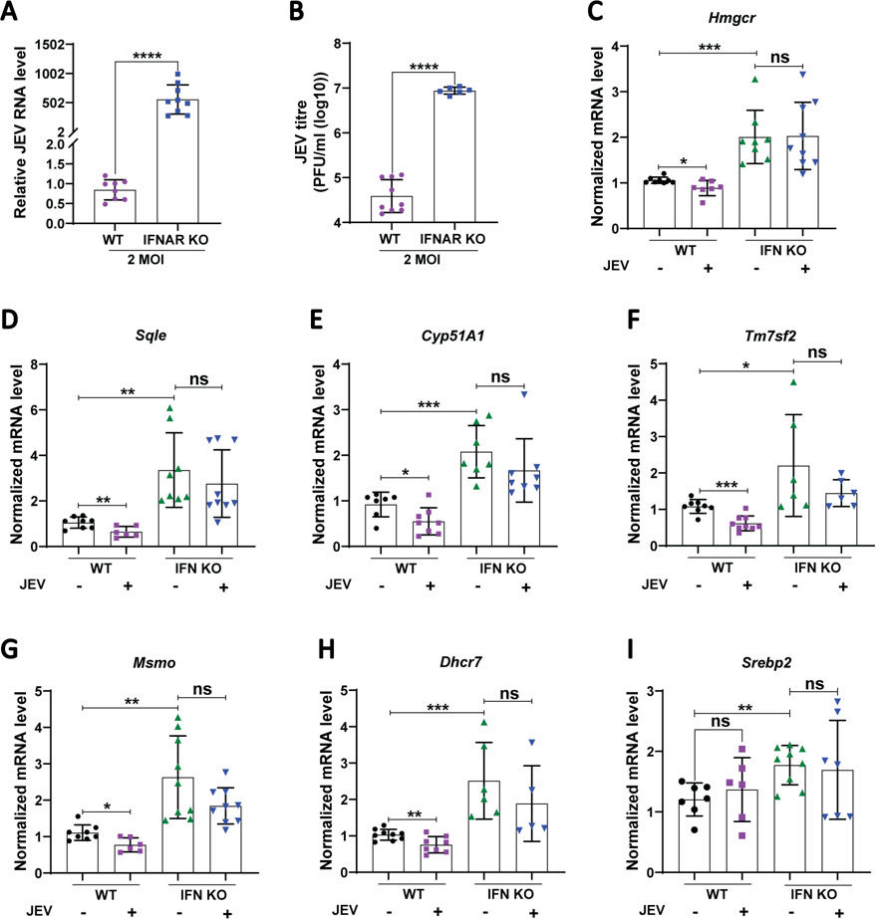
Transcriptional downregulation of cholesterol biosynthetic pathway is dependent upon IFN signaling. WT and IFNAR-/- KO BMDMs were MOCK/JEV (MOI of 2) infected. At 24 hpi, JEV RNA (**A**) and mRNA levels of *Hmgcr* (**C**), *Sqle* (**D**), *Cyp51A1* (**E**), *Tm7sf2* (**F**), *Msmo* (**G**), *Dhcr7* (**H**), and *Srebp2* (**I**) were measured using qRT-PCR and normalized to mock-infected cells. The extracellular virus titer was assessed by plaque assays (**B**). The data presented are the mean ± SD of values obtained from three independent experiments. A Student’s *t*-test was used to calculate *P* values (ns, not significant, *P < 0.05, **P < 0.01, ***P < 0.001, ****P < 0.0001).

Studies have shown that the downregulation of sterol biosynthesis in mouse cytomegalovirus (CMV) infection is dependent on type I IFN signaling (22). To check this in the context of JEV infection, we employed BMDMs from wild-type (WT) and type I and type II IFN receptor (IFN-α/β/γ R-/-) knockout AG129 mice (23). A massive upregulation of virus replication was observed in the AG129 BMDMs, highlighting the enhanced vulnerability of these cells to JEV infection in the absence of IFN signaling (Fig. 3A and B). Interestingly, these cells also showed significantly higher basal mRNA levels of the cholesterol metabolic genes: *Hmgcr*, *Sqle*, *Cyp51A1*, *Tm7sf2*, *Msmo*, *Dhcr7, and Srebp2,* indicating an inverse relation between IFN signaling and cholesterol biosynthetic pathway (Fig. 3C through I). The expression levels of these genes were also compared between JEV-infected WT and IFN-α/β/γ R-/- BMDMs. As reported above, the WT-infected BMDMs downregulated the cholesterol biosynthetic genes; however, the IFN-α/β/γ R-/- BMDMs showed no significant change in the mRNA levels (Fig. 3C through I). This indicates that the JEV-mediated downregulation of the cholesterol biosynthetic pathway requires an active IFN signaling network.

### Depletion of *Dhcr7* inhibits JEV infection and enhances IFN signaling

**Fig 4:**
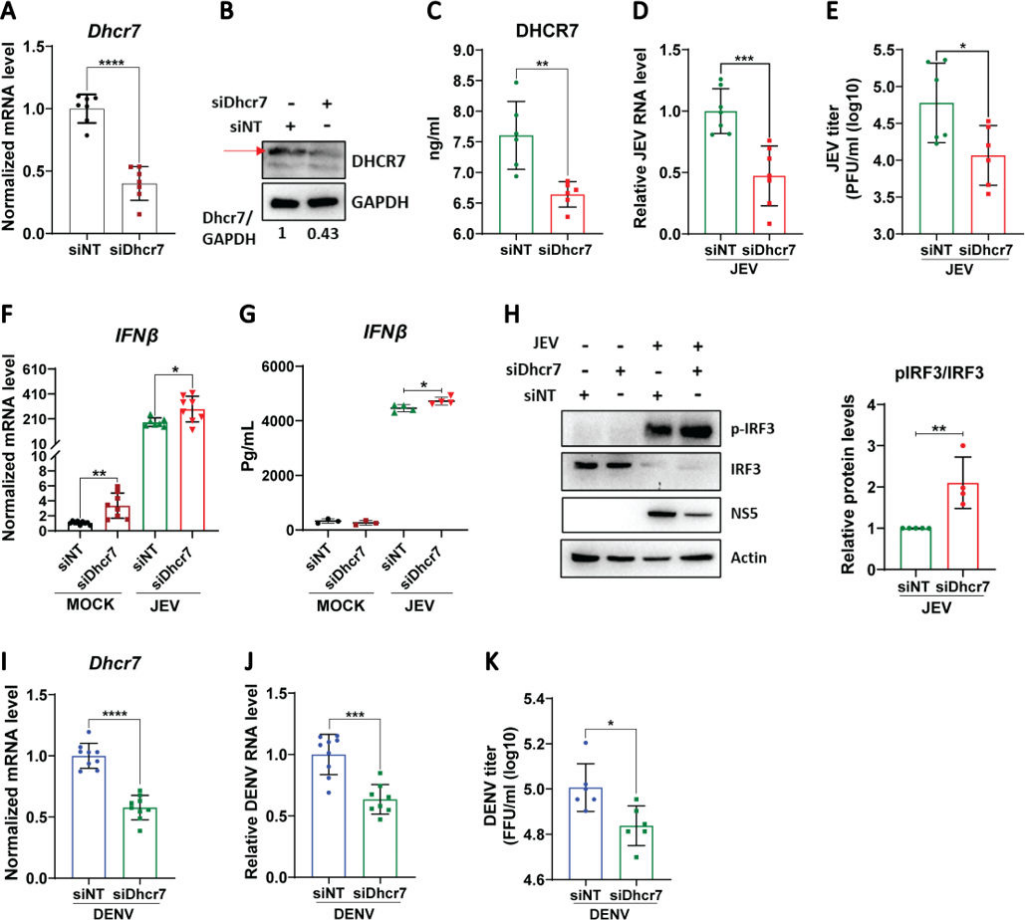
Depletion of *Dhcr7* inhibits JEV infection and enhances interferon signaling. (**A–E**) MEFs were transfected with NT/Dhcr7 siRNA for 48 h, followed by JEV infection at an MOI of 2. At 24 hpi, mRNA levels of *Dhcr7* (**A**) and JEV RNA (**D**) were determined by qRT-PCR (normalized to siNT control), and JEV titers (**E**) were measured by plaque assays. Protein lysates from the same experiment were analyzed by western blotting for DHCR7 and GAPDH (**B**), and DHCR7 levels were measured by ELISA and normalized to siNT (**C**). (**F**) Transcript levels of IFNβ normalized to siNT Mock were determined by qRT-PCR. (**G**) Secretory levels of IFNβ were quantified by CBA. The data presented are the mean ± SD of values obtained from three independent experiments. (**H**) Western blot showing protein levels of pIRF3, IRF3, NS5, and Actin (loading control) analyzed from cell lysates of siNT/Dhcr7-transfected and JEV-infected MEFs. Bar graph on the right shows quantification of pIRF3/IRF3 ratio from four independent experiments. (**I–K**) siNT/siDhcr7-transfected MEFs were infected with DENV (MOI of 2). At 48 hpi, DENV RNA levels normalized to siNT control (**J**) and DENV FFU (**K**) were determined. A Student’s *t*-test was used to calculate *P* values (*P < 0.05, **P < 0.01, ***P < 0.001, ****P < 0.0001).

Since the *Dhcr7* gene displayed the maximal reduction in virus-infected cells, we examined its functional role during JEV infection. Studies have shown that DHCR7 deficiency can exert antiviral effect through enhancement of IRF3 phosphorylation (24). *Dhcr7* depletion through siRNA targeting in MEFs (Fig. 4A through C) further inhibited JEV replication, as seen by reduced RNA levels (Fig. 4D) and viral titers (Fig. 4E). IFNβ transcripts were increased at basal levels following *Dhcr7* depletion (Fig. 4F), and virus-infected cells also showed higher transcript and secretory levels of IFNβ (Fig. 4F and G). In accordance with earlier studies (24–27), we also observed an increase in IRF3 phosphorylation following Dhcr7 depletion, as indicated by the elevated pIRF3/IRF3 ratio (Fig. 4H). This suggests that depletion of *Dhcr7* can further downmodulate JEV infection and enhance interferon signaling. Inhibition of DENV-2 infection was also observed in *Dhcr7*-silenced cells (Fig. 4I through K).

### Pharmacological inhibition of Dhcr7 with AY9944 restricts JEV infection and enhances IFN signaling

**Fig 5:**
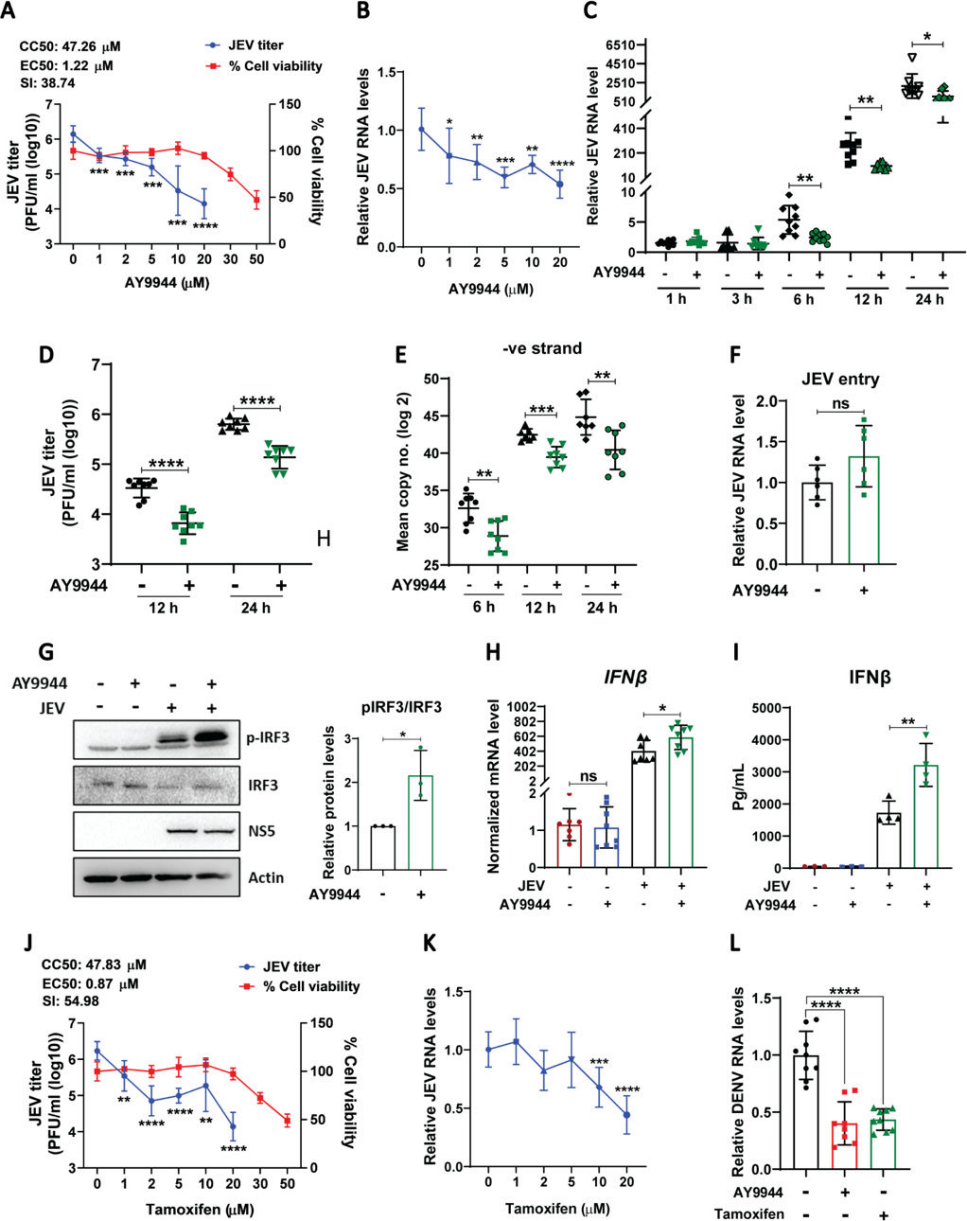
Pharmacological inhibition of Dhcr7 with AY9944 restricts JEV infection and enhances IFN signaling in MEFs. (**A–E**) MEFs were treated with DMSO or AY9944 at the indicated concentrations for 24 h, and cell viability was assessed by MTT assay and plotted after normalization to untreated controls (**A**, right axis). In a separate experiment, cells were infected with JEV at an MOI of 2 and treated with DMSO or AY9944 (1, 2, 5, 10, or 20 µM) at 1 hpi. At 24 hpi, the cells were harvested, and JEV RNA levels normalized to infected controls (**B**, qRT-PCR) and JEV titers (**A**, left axis, plaque assay) were determined. CC50 and EC50 values were calculated by nonlinear regression curve fit using GraphPad Prism. For time course analysis, JEV RNA levels (normalized to mock 1 hpi) (**C**), and titers (**D**) were determined at the indicated time points. (**E**) Negative-strand of viral RNA was quantified using qRT-PCR. (**F**) To assess viral entry, MEFs were infected with JEV at an MOI of 2 on ice for 1 h, then treated with DMSO/AY9944, and incubated at 37°C for 1 h. At 1 hpi, the cells were trypsinized to remove bound virus particles, and the level of internalized virus was measured using qRT-PCR and plotted after normalization to infected controls. (**G**) Western blot shows the protein expression of pIRF3, IRF3, NS5, and Actin (loading control) in cell lysates from JEV-infected MEFs treated with DMSO/AY9944. Bar graph on the right showing quantification of pIRF3/IRF3 ratio normalized to infected control from three independent experiments. (**H and I**) Graphs showing IFN-β transcript levels (**H**, qRT-PCR, normalized to mock) and IFN-β-secreted levels (**I**, ELISA). (**J and K**) MEFs were treated with DMSO or tamoxifen at the indicated concentrations for 24 h, and cell viability was assessed by MTT assay and plotted after normalization to untreated controls (**J**, right axis). For infection experiments, MEFs were infected with JEV at an MOI of 2 and treated with tamoxifen (1, 2, 5, 10, or 20 µM). Viral RNA levels (**K**) were quantified, and viral titers (**J**, left axis) were determined by plaque assay. CC50 and EC50 values were calculated by nonlinear regression curve fit using GraphPad Prism. (**L**) MEFs were treated with AY9944 (10 µM) and tamoxifen (10 µM) following DENV infection, and viral RNA levels normalized to infected controls were plotted. The data presented are the mean ± SD of values obtained from three independent experiments. A Student’s *t*-test was used to calculate *P* values (ns, not significant, *P < 0.05; **P < 0.01, ***P < 0.001, ****P < 0.0001).

To assess the impact of inhibiting the enzymatic activity of Dhcr7, we used its selective inhibitor, AY9944, which blocks cholesterol biosynthesis by preventing the conversion of 7DHC to cholesterol (28). Treatment with AY9944 significantly reduced JEV replication with an EC50 of 1.22 µM (Fig. 5A and B). To determine the specific stage of the viral life cycle affected by AY9944, we performed a time-course analysis. Viral RNA levels remained identical between control and AY9944-treated cells up to 1–3 h post-infection (hpi), suggesting that AY9944 does not affect the early stages of the viral life cycle. Once the viral RNA is translated, replication complexes assemble on the ER membrane, where plus-strand RNA is reverse-transcribed into a negative strand, generating dsRNA replicative intermediates. This process is preceded by a rapid surge in plus-strand RNA (29, 30). AY9944 inhibited virus replication starting at 6 hpi, along with a corresponding decrease in the copy number of negative-strand RNA (Fig. 5C through E). Additionally, no significant change was observed in the JEV entry following the AY9944 treatment (Fig. 5F). AY9944 treatment also activated IRF3 (Fig. 5G), along with upregulation in IFNβ transcript and secretory levels (Fig. 5H and I). These results suggested that, like Dhcr7 depletion, pharmacological inhibition of Dhcr7 enzyme activity also enhances the IFN activation and inhibits virus replication. We also tested another Dhcr7 inhibitor, tamoxifen, and observed reduction in JEV replication (Fig. 5J and K), further confirming the potential of Dhcr7 inhibition as an antiviral strategy for JEV. Both these drugs also significantly inhibited DENV-2 replication (Fig. 5L).

**Fig 6:**
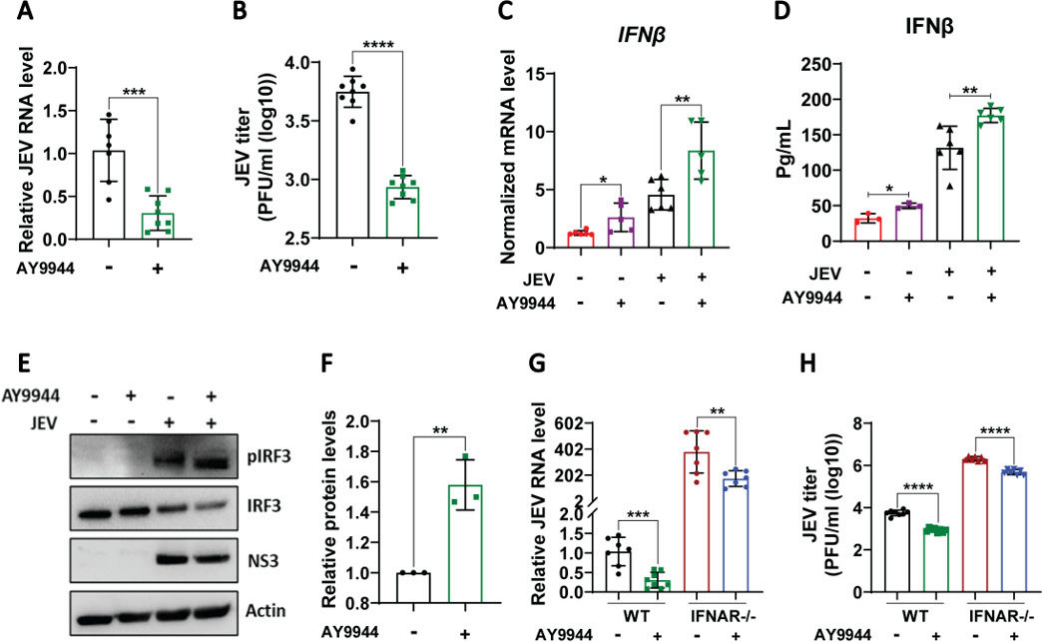
Pharmacological inhibition of Dhcr7 with AY9944 restricts JEV infection and enhances IFN signaling in BMDMs. (**A and B**) BMDMs were infected with JEV (MOI of 2, 1 h). Following infection, cells were treated with DMSO or AY9944 (10 μM) for 24 h. At 24 hpi, the viral transcript levels (**A**) were quantified using qRT-PCR (normalized to infected controls), and viral titers (**B**) were determined via plaque assays. (**C and D**) The bar graph represents IFNβ mRNA levels (**C**) measured by qRT-PCR (normalized to uninfected controls), and secreted IFNβ levels (**D**) were measured using ELISA. (**E**) Western blot showing the protein expression of pIRF3, IRF3, NS3, and Actin (loading control) in the cell lysates of DMSO/AY9944 treated JEV-infected BMDMs. (**F**) Bar graph shows the pIRF3/IRF3 ratio normalized to infected controls from three independent experiments. (**G and H**) WT and IFNAR⁻/⁻ KO BMDMs were treated with DMSO (control) or AY9944 after infection at an MOI of 2. The graph represents JEV transcript levels (**G**, qRT-PCR, normalized to infected controls) and viral titers (**G**, plaque assay). The data presented are the mean ± SD of values obtained from three independent experiments. A Student’s *t*-test was used to calculate the *P* values (*P < 0.05; **P < 0.01, ***P < 0.001, ****P < 0.0001).

The impact of AY9944 treatment was also checked in BMDMs, where significant inhibition of virus replication (Fig. 6A and B) and enhancement in both the transcript and secretory levels of IFNβ (Fig. 6C and D), along with higher IRF3 phosphorylation (Fig. 6E and F), were observed. To determine whether AY9944’s antiviral activity relies on IFN signaling, we tested its effect in IFN-α/β/γ R⁻/⁻ BMDMs. Interestingly, the AY9944 treatment in IFN-α/β/γ R⁻/⁻ BMDMs also reduced virus replication, but to a lesser extent as compared to WT BMDMs (Fig. 6G and H). These data suggest that while the enhancement of IFN signaling is the key driver of the observed effects of Dhcr7 inhibition, some IFN-independent antiviral effects are also operational.

### Supplementation of 7-dehydrocholesterol exerts an antiviral effect

**Fig 7:**
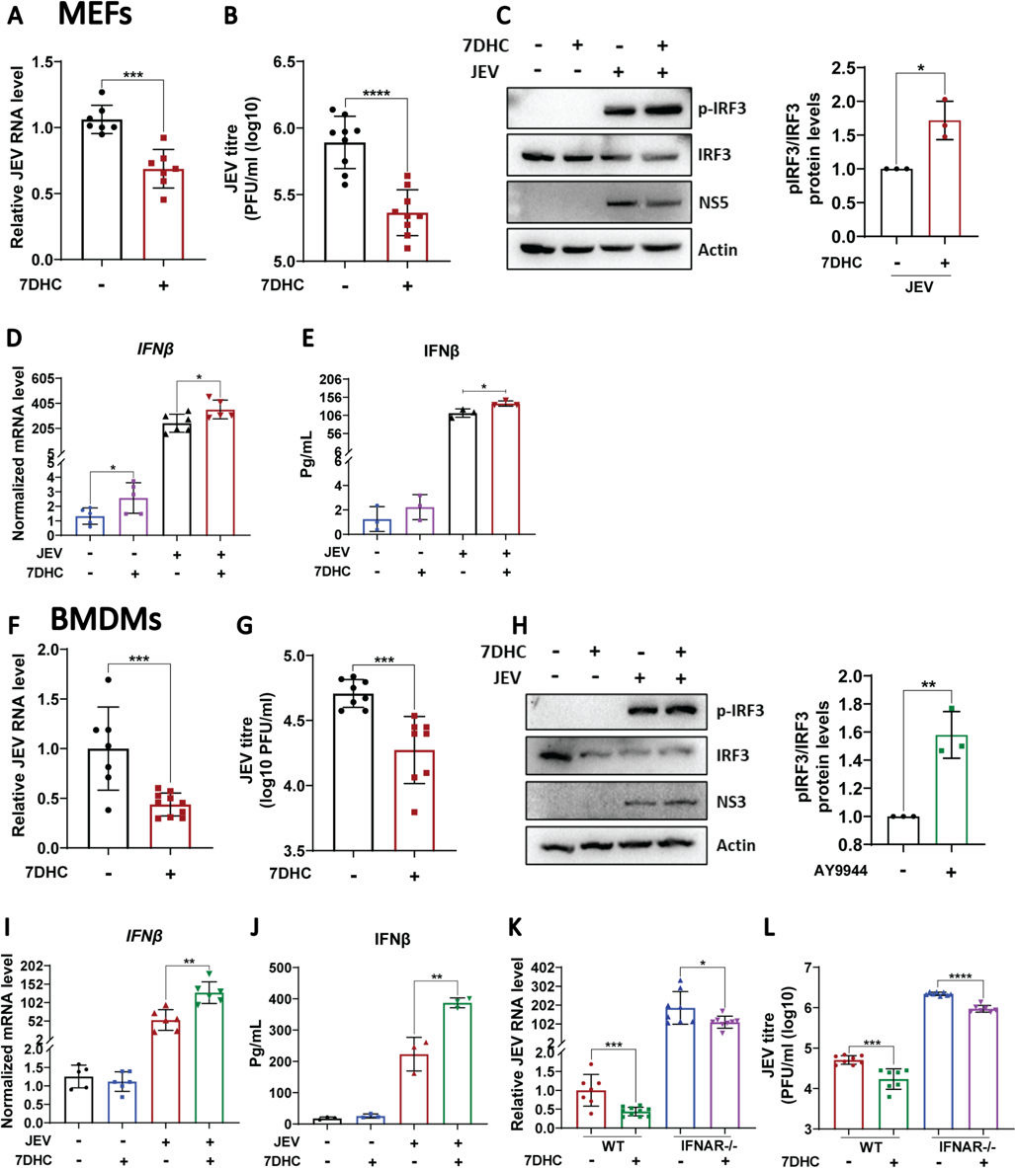
Supplementation of 7DHC exerts an antiviral effect. (**A–E**) MEFs were infected with JEV at an MOI of 2. At 1 hpi, cells were treated with Ethanol/7DHC (10 uM). At 24 hpi, JEV RNA levels normalized to infected controls (**A**) were determined using qRT-PCR, and JEV titer was assessed by plaque assay (**B**). (**C**) Western blot showing the protein expression of p-IRF3, IRF3, NS5, and Actin (loading control) in the cell lysates of Ethanol/7DHC-treated JEV-infected MEFs. Bar graph (right panel) showing quantification of pIRF3/IRF3 ratio from three independent experiments. (**D**) IFNβ mRNA levels were measured using qRT-PCR (normalized to mock-infected controls). (**E**) IFNβ secretion was analyzed in the cell supernatants by ELISA (**E**). (**F and J**) BMDMs were infected with JEV at an MOI of 2. At 1 hpi, the cells were treated with Ethanol/7DHC (10 uM). At 24 hpi, JEV RNA (F, qRT-PCR, normalized to control) and titer (**G**, plaque assay) were assessed. (**H**) The protein expression of pIRF3, IRF3, NS3, Actin (loading control) was analyzed in the cell lysates by western blotting. The bar graph (**H**, right panel) depicts the pIRF3/IRF3 ratio normalized to infected controls from three independent experiments. (**I**) IFNβ mRNA levels were measured using qRT-PCR (normalized to mock). (**J**) IFNβ secretion was analyzed in the cell supernatants by ELISA. (**K and L**) WT and IFNAR-/- KO BMDMs were infected with JEV at an MOI of 2 for 1 h, followed by 7DHC addition. At 24 hpi, JEV RNA (**K**, qRT-PCR) and JEV titer (**L**, plaque assay) were determined. The data presented are the mean ± SD of values obtained from three independent experiments. A Student’s *t*-test was used to calculate the *P* values (*P < 0.05, **P < 0.01, ***P < 0.001, ****P < 0.0001).

7DHC, the substrate of the *Dhcr7* gene, accumulates following its depletion. Studies have reported that like *Dhcr7* inhibition, 7DHC also limits viral infection and causes an increase in the interferon response (24). The exogenous addition of 7DHC significantly reduced virus replication as observed by JEV RNA levels and titers in MEFs and BMDMs (Fig. 7A and B, F through G). 7DHC treatment also significantly enhanced the pIRF3 levels (Fig. 7C and H), and the IFNβ transcript and secretory levels (Fig. 7D and E, I and J). In the IFN-α/β/γ R-/- BMDMs, 7DHC supplementation showed some inhibition of JEV replication (Fig. 7K and L), highlighting the existence of an IFN-independent antiviral effect of modulating Dhcr7.

### Antiviral effect of Dhcr7 inhibition is mediated by 7DHC accumulation

**Fig 8:**
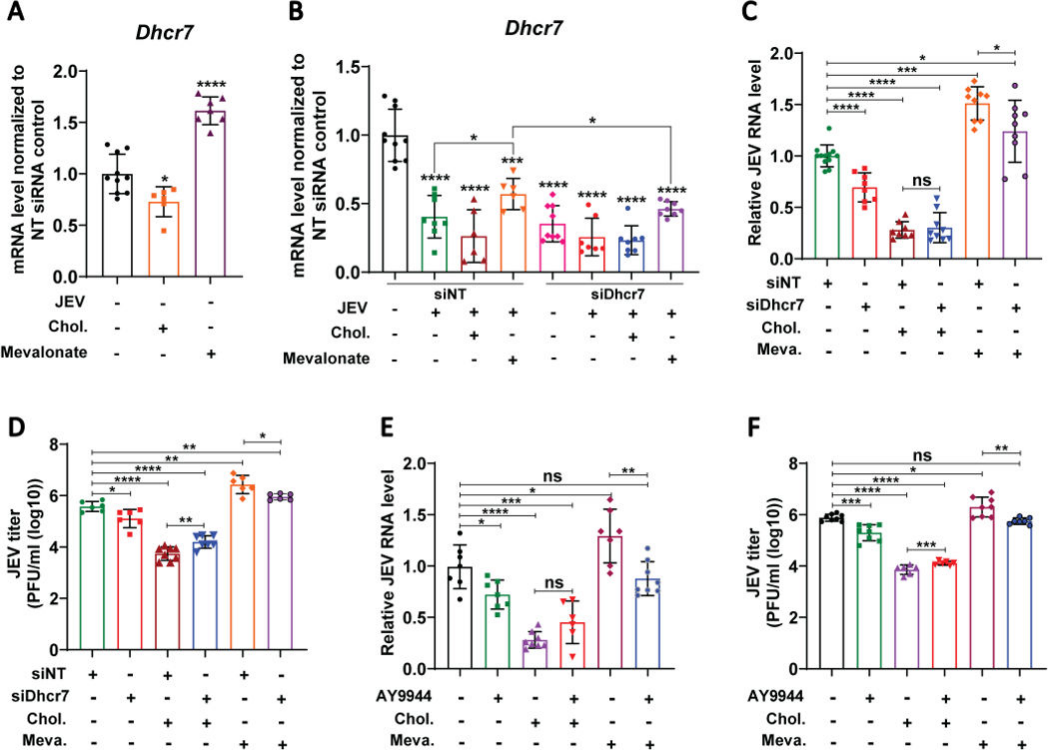
Antiviral effect of Dhcr7 inhibition is mediated by 7DHC accumulation. (**A–C**) MEFs were transfected with NT/Dhcr7 siRNA for 48 h, followed by MOCK/JEV infection (MOI of 1) and treatment with cholesterol (20 µg/mL) and mevalonate (100 µM) for 24 h. Dhcr7 mRNA levels (**A and B**) and JEV RNA levels normalized to siNT control (**C**) were measured by qRT-PCR. (**D**) Virus titer was determined using the plaque assay. (**E and F**) Representative graphs depict JEV RNA levels (normalized to infected controls) (**E**) and titers (**F**) at 24 hpi in MEFs treated with AY9944 (10 µM), cholesterol (20 µg/mL), and mevalonate (100 µM) following infection with JEV at an MOI of 2. The data presented are the mean ± SD of values obtained from three independent experiments. A Student’s *t*-test was used to calculate the *P* values (ns, not significant, *P < 0.05, **P < 0.01, ***P < 0.001, ****P < 0.0001).

The metabolite 7-DHC accumulates following the depletion or inhibition of *Dhcr7*. We next tested whether supplementation with metabolites either upstream or downstream of Dhcr7 enzymatic activity could influence its antiviral effect. For this, we exogenously added cholesterol (the end product) and mevalonate (an upstream precursor) to cells. Under mock conditions, cholesterol supplementation significantly reduced *Dhcr7* mRNA levels, while mevalonate supplementation enhanced it (Fig. 8A). In siNT JEV-infected cells, as expected, *Dhcr7* mRNA levels were lower and were further reduced by cholesterol supplementation but enhanced by mevalonate (Fig. 8B). Treatment with siDhcr7 significantly reduced the mRNA levels under all conditions (Fig. 8B). Cholesterol supplementation during JEV infection reduced viral replication in both siNT- and siDhcr7-treated cells (Fig. 8C and D). This is consistent with reports wherein supplementation or depletion of cholesterol reduces flavivirus replication (3). In contrast, mevalonate supplementation in siNT-treated cells enhanced viral replication, likely through upregulation of the cholesterol biosynthetic pathway genes including *Dhcr7*. Depletion of Dhcr7 through siRNA was able to inhibit virus replication under conditions of mevalonate supplementation, underscoring the antiviral potential of Dhcr7 inhibition (Fig. 8C and D). Similar results were obtained with AY9944 treatment (Fig. 8E and F). These results suggest that the downregulation or inhibition of Dhcr7 is essential for exerting an antiviral effect.

## DISCUSSION

Lipid metabolic pathways and host lipids play a crucial role in determining virus pathogenesis and are often subverted by viruses to enhance their replication (31). Using a metabolomics analysis approach, studies have shown the dysregulation of lipid biosynthetic processes upon infection by different viruses including flaviviruses (32–35). This shift in the lipid metabolic profile is critical for remodeling cellular membranes, enabling the formation of viral replication complexes (36–39). In addition, the processes of attachment, entry, and egress of flaviviruses, which involve crossing the plasma membrane, are also reported to be influenced by lipids (1–5). Our previously published omics studies have strongly indicated that dysregulation of lipid metabolic pathways is an inherent feature of JEV infection (20, 40). The consequences of virus-induced lipid metabolic rewiring are multifaceted, ranging from the formation of virus replication complexes to modulation of innate immune responses and inflammation (22). This provides a strong rationale for exploring how JEV alters host lipid metabolism, which may offer novel opportunities to combat JEV pathogenesis through targeted manipulation of metabolic pathways.

Here, we identified a total of 840 lipid species, mainly belonging to carnitines, sterols, sphingolipids, phospholipids, glycerolipids, ceramides, sphingomyelins, and plasmalogens. JEV infection dysregulated approximately 41% of the total identified lipidome. Major changes were seen in the abundance of sterols, sphingolipids, phospholipids, glycerolipids, and plasmalogens. Among the phospholipid subclasses, PC, PS, PI, and PG, as well as ceramides and plasmalogens (except plasmanyl-TG), were found to be increased in the infected cells, whereas the levels of sterols, glycerolipids, and plasmanyl-TG were reduced during infection (listed in Data set S1). Further analysis of cholesterol biosynthesis pathway genes revealed a transcriptional downregulation of their expression, where Dhcr7 showed the most pronounced reduction of nearly 50% in virus-infected MEFs and BMDMs, suggesting that JEV infection results in a coordinated downregulation of the cholesterol biosynthetic pathway and a decrease in cholesterol levels in infected cells.

Viral infections and the activation of interferon signaling have been reported to suppress cholesterol biosynthesis by inhibiting SREBP2 activity (13, 22). While viruses manipulate cholesterol metabolism to support their replication, these metabolic alterations can also benefit the host by enhancing antiviral immune responses, particularly through the activation of interferon signaling in infected cells (13–17). Selective reduction of the synthesized pool of cholesterol has been shown to spontaneously activate type I IFN signaling, thereby priming cells for an antiviral state (13). Furthermore, cholesterol inhibition using statins has been found to enhance RIG-I-mediated antiviral responses against Sendai virus and increase the production of non-canonical type I interferons, such as IFN-ω and IFN-α16 (18). In our study, like the effects observed during JEV infection, both IFN-γ stimulation and activation of the toll-like receptor (TLR) pathway led to the downregulation of genes involved in cholesterol biosynthesis. Importantly, we found that this downregulation was interferon-dependent, as there was no change in the expression of cholesterol pathway genes in JEV-infected IFN-α/β/γ R−/− BMDMs. These findings suggest that JEV-mediated suppression of cholesterol biosynthesis requires an active interferon signaling pathway.

**Fig 9:**
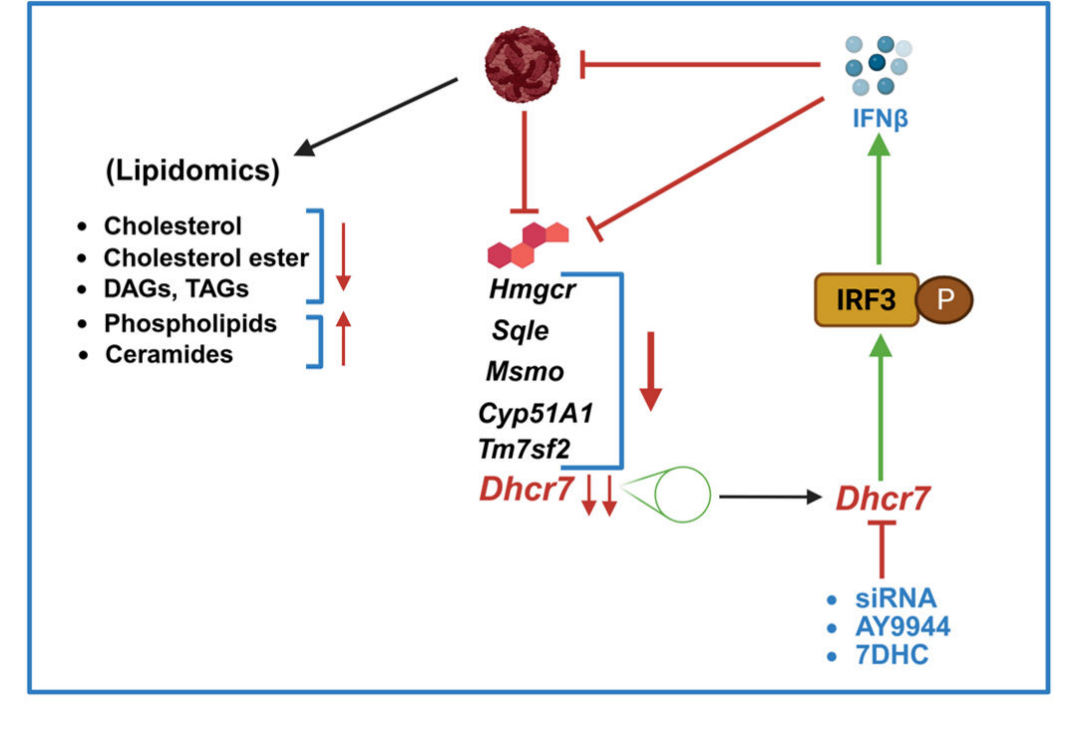
Schematic model. JEV infection reduces the levels of cholesterol, cholesterol esters, and glycerolipids, while increasing the levels of ceramides and various phospholipids in the lipidomic data. This is accompanied by transcriptional downregulation of genes involved in cholesterol biosynthesis. siRNA-mediated knockdown and pharmacological inhibition of Dhcr7 using AY9944, as well as supplementation with 7DHC, enhance the phosphorylation of IRF3, thereby activating IFN-β signaling. This leads to interferon-induced suppression of both JEV replication and cholesterol biosynthesis genes. Created with Biorender.com*.*

Viral infections manipulate host cell pathways involved in cholesterol uptake and synthesis, and inhibiting these pathways has been reported to have antiviral effects against several viruses, including DENV, WNV, and Chikungunya virus (CHIKV) (9–12, 41). Studies have shown that DENV infection leads to increased enzymatic activity of *HMGCR* and that inhibition of HMGCR reduces active viral replication (11, 42). Similarly, knockdown of the *MVD*, which is involved in the cholesterol biosynthesis pathway, reduced DENV infection (10). Furthermore, infections with Zika virus (ZIKV) and pseudorabies virus (PRV) have been shown to upregulate *Dhcr7*, an enzyme essential for the final step of cholesterol biosynthesis. Inhibition of *Dhcr7* effectively reduces replication of both ZIKV and PRV (25, 26). Mechanistically, *Dhcr7* promotes viral infection by suppressing the phosphorylation of IRF3, which in turn lowers IFN-β production and diminishes the induction of interferon-stimulated genes (ISGs). In contrast, an intriguing study reported that infections by various RNA and DNA viruses downregulate *Dhcr7* expression in macrophages (24). Both genetic deletion and pharmacological inhibition of *Dhcr7* significantly impaired ZIKV replication *in vitro* and *in vivo*. Moreover, *Dhcr7* deficiency or treatment with its substrate, 7-dehydrocholesterol, enhanced type I interferon signaling in macrophages. An antiviral strategy utilizing polymerized coronavirus receptors (COVR-MV) was also shown to reduce infection by highly pathogenic coronaviruses by enhancing type I interferon responses via *Dhcr7* inhibition (27). Additionally, three Food and Drug Administration-approved *Dhcr7* inhibitors, cariprazine, trazodone, and ifenprodil, along with the commonly used inhibitor AY9944, inhibited GFP-tagged vesicular stomatitis virus (VSV) in a dose-dependent manner (43). All these studies indicate that the inhibition of Dhcr7 is associated with enhanced innate immunity and could be explored as a potential antiviral strategy. In our study, we found that both knockdown and pharmacological inhibition of *Dhcr7* exerted a potent antiviral effect against JEV by blocking viral replication and enhancing interferon signaling. These findings suggest that while enhanced IFN signaling is the key driver of the antiviral effects observed with *Dhcr7* inhibition, IFN-independent mechanisms may also contribute. Similarly, direct supplementation with 7DHC showed antiviral effects comparable to those observed with *Dhcr7* inhibition, likely acting through a similar mechanism. This further highlights the potential of *Dhcr7* as an antiviral target against JEV (Fig. 9).

Published studies have shown that the inhibitory effect of *Dhcr7* knockdown or AY9944 treatment on enterovirus A71 (EV-A71) replication can be reversed by cholesterol supplementation (44). Additionally, *Dhcr7* has been reported to support pseudorabies virus (PRV) replication by converting 7DHC into cholesterol, thereby increasing cellular cholesterol levels (26). Surprisingly, our results showed that cholesterol supplementation strongly inhibited viral RNA levels and titers, suggesting that cholesterol overloading exerts antiviral effects against JEV. Previous studies have reported that both the addition and inhibition of cholesterol reduce JEV and DENV replication, primarily by blocking viral entry. These findings highlight that precise cholesterol levels in target membranes are critical for viral entry, and that either increasing or decreasing cholesterol levels can disrupt this balance (3). Furthermore, we observed that mevalonate supplementation enhanced or restored JEV replication. Existing literature suggests that the mevalonate pathway supports viral infection through the production of geranylgeranyl pyrophosphate (GGPP), rather than cholesterol itself (10, 45, 46). In our study, cholesterol and mevalonate supplementation under *Dhcr7*-depleted or AY9944-treated conditions resulted in reduced JEV replication, indicating that the observed antiviral effects may be primarily mediated through the accumulation of 7-dehydrocholesterol.

In summary, our study highlights an inverse interplay between cholesterol biosynthetic pathway and IFN signaling during JEV infection, and an operational IFN-independent antiviral mechanism, providing a foundation for exploring this axis as an antiviral target for JEV.

## MATERIALS AND METHODS

### Cells and virus

Mouse embryonic fibroblasts (MEFs) were procured from the RIKEN Bio-Resource Cell Bank (Cat. No. RCB2710). The cell lines C6/36 (insect) and Vero were obtained from the National Centre for Cell Sciences in Pune, India. All cell lines were negative for mycoplasma. MEFs were cultured in Dulbecco’s modified Eagle’s medium (DMEM), and Vero and C6/36 were cultured in Eagle’s minimum essential media (MEM). The media was supplemented with additional constituents such as 10% fetal bovine serum (FBS), 100 µg/mL penicillin-streptomycin combination, and 2 mM L-glutamine.

JEV genotype 3, P20778 strain (GenBank accession AF080251), and DENV-2 strain P23085 INDI-60 (GenBank accession KJ918750) were generated in C6/36. For the mice studies, the JEV S3 strain (mouse-adapted) was used (47). The plaque/foci-forming units (FFU) assay for virus titer determination was done in Vero cells. All antibodies, reagents, and drugs used in the study are listed in Table S1.

C57BL/6 and AG129 mice 6–8 weeks old were used for BMDM isolation. Mice were euthanized, and femurs and tibias were dissected, washed with PBS, and flushed with RPMI supplemented with L929-conditioned medium to extrude bone marrow. Red blood cells (RBCs) were lysed, and the remaining cells were cultured in RPMI complete media supplemented with L929-conditioned media for 7 days. BMDMs were detached using 10 mM EDTA and seeded in 24-well plates for virus infection and drug treatment experiments.

### Lipid extraction

Lipid extraction from MEFs was performed using the protocol described in (48). Cells (10^7) were mixed with 100 µL of methanol by brief vortexing and sonication in a water bath for 10 min. An additional 0.3 mL of methanol was added to the sample and vortexed for 30 s. After adding 1.25 mL MTBE (methyl-tert-butyl ether), the sample mixture was incubated on a shaker for 1 h at RT. Later, 0.3 mL of MS-grade water was added, and the sample was incubated at RT for 10 min. This step induces the phase separation. The sample phases were separated at 400 rpm for 5 min at 10°C. The upper organic phase was collected, dried in a speed vac, and stored in −80°C till use. The lipid extract was dissolved in 100 µL of 65:30:5 (acetonitrile:2-propanol:water, vol/vol/vol) solvent when required.

### Lipid measurement

Lipids were separated using Acquity HSS T3 (2.1 mm × 100mm × 1.8 Um, Waters), ultra-performance liquid chromatography (UPLC). For UPLC, water/acetonitrile (2:3 vol/vol) was used as solvent A, and 2-propanol/acetonitrile (9:1, vol/vol) was used as solvent B, the flow rate was kept at 0.3 mL/min, and the column temperature was set at 40°C. UPLC was run for 18 min with the following gradient setup: for 0–12 min, the amount/concentration of solvent B was increased from 30% to 97%, followed by a hold of 3 min. During the 15.2–18 min run, solvent B was maintained at 30%. Data were acquired on a high-resolution mass spectrometer, orbitrap fusion (Thermo Scientific), equipped with a heated electrospray ionization source (ESI). ESI sheath and auxiliary gas flow were used at 60 and 20 (arbitrary units). Positive and negative spray voltage was set at 3,000 volts. For the entire MS run, the setup of 120 k resolution with automatic gain control (AGC) target of 200,000 and mass ranges between 250 and 1,200 was used. For MSMS, 30 k resolution with an AGC target of 50,000 was used. A collision energy of 27+/−3 was used for fragmentation.

### Lipid data analysis

Lipidmatch flow was used for peak picking (using mzmine), blank filtration, lipid annotation, and combining positive and negative data (49). Statistical analysis of raw files of LC-MS data were done using MetaboAnalyst software (50). Data were normalized by sum, operator-scaled, and log-transformed for analysis in MetaboAnalyst software. Lipid species with ≥ −1 and ≥ 1 log2 (FC) and *P* <0.05 were selected for further study.

### Virus infection and cell treatment

The respective cell types were washed and infected with JEV/DENV-2 at different MOIs (1, 2, 5, and 10) for 1 h for virus infection experiments. Following infection, the cells were washed, and complete media was added. Drug treatment was given by adding 1, 2, 5, 10, or 20 µM AY9944 or tamoxifen, after which the cells were harvested at the indicated time points and processed for RNA isolation and cell lysate preparation. Cell culture supernatants were used to quantify viral titers and inflammatory cytokines via ELISA. The 50% effective concentration (EC50) was determined from viral titers using nonlinear regression curve fit in GraphPad Prism. For 50% cytotoxic concentration (CC50) determination, cells were treated with various concentrations of AY9944 and Tamoxifen for 24 h, after which cell viability was assessed using the 3-(4,5-dimethylthiazol-2-yl)−2,5-diphenyltetrazolium bromide (MTT) assay. The selectivity index (SI) was calculated as CC50 divided by EC50. IFN-γ was administered at 30 ng/mL, LPS at 1 µg/mL, and DMXAA at 200 µg for the indicated durations. MEFs were transfected with poly I:C (1 µg/mL) using Lipofectamine P3000 for 6 h. All experiments included biological triplicates and were performed at least twice.

### RNA isolation and quantitative real-time PCR (qRT)-PCR

Total RNA was manually extracted from cells using the RNAiso reagent (#9109, Takara). cDNA was synthesized from the extracted RNA using random hexamers and the GoScript Reverse Transcriptase kit (A5001, Promega). qRT-PCR reactions were set up on the QuantStudio 6 (Applied Biosystems) using SYBR Premix Ex Taq II (RR420) and Premix Ex Taq II mix (RR390A, Takara Bio). JEV RNA levels were quantified using specific primers and TaqMan probes, while GAPDH served as an internal housekeeping control. Gene expression analysis of the cholesterol, interferon, and inflammatory pathways was performed using SYBR mix reagents, and fold changes were calculated after normalization to mock/DMSO controls. Each experiment included biological and technical triplicates and was performed thrice. The primer sequences for all genes analyzed in this study are listed in Table S2.

### Quantitative analysis of negative-sense JEV RNA strand

Following a previously published protocol, the negative-sense JEV RNA was quantified using qRT-PCR through a strand-specific assay (51). Briefly, total RNA was extracted from JEV-infected MEFs (MOI of 2) treated with either DMSO (control) or 10 µM AY9944, then processed for strand-specific qRT-PCR. First, cDNA was synthesized using a tagged (non-viral sequence) reverse transcription primer (NVnegVneg, Table S2). qRT-PCR was then performed to specifically assess the expression of the capsid gene (Table S2) using a TaqMan probe (Negative sense probe). The qPCR reaction included a combination of primers—one binding to the non-viral tag sequence (qPCR-F) and the other to the viral strand (qPCR-R) (Table S2), along with the Premix Ex Taq (Probe qPCR) master mix. A standard curve was generated using an *in vitro*-transcribed negative-strand RNA of the JEV capsid gene to determine absolute negative-sense JEV RNA quantities. This curve established a correlation between the copy number of negative-strand JEV RNA and the Ct values obtained from RNA concentrations (g/µL) (52).

### Dhcr7 knockdown

For the Dhcr7 knockdown experiment, MEFs were transfected with either mouse-specific Dhcr7 siRNA or non-targeting (NT) siRNA (30 nM, ON-TARGET plus SMART pool) using Lipofectamine RNAiMAX. After 48 h post-transfection, the cells were harvested, and knockdown efficiency was assessed using qRT-PCR, western blotting, and Dhcr7 ELISA. For infection experiments, 48 h after transfection with NT or Dhcr7 siRNA, the cells were infected with JEV/DENV-2 at an MOI of 2. Cells were then harvested at specific time points post-infection and processed for viral RNA quantification.

### Cell fractionation

MEFs were mock- or JEV-infected at an MOI of 2. At 24 hpi, cells were trypsinized and washed with 1× PBS and resuspended in 500 µL of cytoplasmic buffer. The samples were incubated on ice for 10–15 min. A 250 µL aliquot was collected as the “whole-cell extract” and stored at −80°C for future use. The remaining sample was centrifuged at 10,000 rpm for 30 s at 4°C, and the supernatant was transferred to new microcentrifuge tubes (MCTs). The centrifugation step was repeated once, and ~ 200 µL of the supernatant was collected and labeled as the “cytoplasmic extract.” Protein concentrations in the samples were determined using a BCA assay. The required volume of 5× loading dye was added, and samples were heated at 95°C for 10 min before being stored at −20°C for subsequent western blot analysis. The pellet remaining in the original MCT was labeled as the “nuclear extract.” It was washed with nuclear buffer by incubating for 5 min, followed by a short centrifugation at 10,000 rpm for 30 s at 4°C. The supernatant was carefully aspirated to avoid disturbing the pellet. This washing step was repeated 2–3 times. Finally, the pellet was resuspended in 50 µL of 5× loading dye and heated at 95°C for 20 min, with gentle vortexing every 5 min.

### Western blot analysis

MEFs/BMDMs were washed with 1× PBS and lysed in a lysis buffer containing 20 mM Tris-HCl, 1 mM EDTA, 250 mM NaCl, 1% Triton X-100, 1 mM PMSF, and 1 mM protease inhibitor. For cell lysate preparation, the cells were lysed on ice with continuous vortexing every 15 min for approximately 1 h. The lysate was then centrifuged at 15,000 × *g* for 10 min at 4°C. The protein concentration in the post-centrifugation cell lysate was determined using the Pierce BCA assay kit (Cat. No. 23225). The lysate was subsequently heated at 95°C for 10 min in 1× Laemmli buffer to denature proteins. Equal concentrations of proteins were separated based on molecular weight via SDS-PAGE, followed by transfer onto PVDF membranes. After transfer, the membranes were blocked with 5% skimmed milk for 1 h at RT. They were then incubated overnight at 4°C with the desired antibodies prepared in 5% BSA (Sigma, A7906). The blots were developed using a Gel Doc XR+ gel documentation system (Bio-Rad), and protein fold changes were calculated by measuring band intensities using ImageJ (NIH, USA) software. All western blotting experiments were performed at least three times, and representative blots are shown.

### Virus titration by plaque assay

The infectivity of the virus in the cell supernatants was determined using a plaque assay. In brief, Vero cells (1.8 × 10⁵ cells per well) were seeded in 12-well plates and incubated for at least 24 h to form a monolayer. In total, 10-fold serial dilutions of the virus stock were prepared in incomplete MEM, and the Vero cell monolayer was infected for 1 h at 37°C with gentle rocking. After 1 h, the virus inoculum was removed, and the cells were washed with PBS. The monolayer was then overlaid with a 1:1 mixture of complete MEM and 1% low-melting point agarose (Agarose Type VII, Sigma, A4018). The cells were incubated at 37°C for 5 days until plaques became visible. Following the 5-day incubation, the cells were fixed overnight with 3.7% formaldehyde and then stained with 0.1% crystal violet to visualize the plaques.

### Cytokine bead array (CBA)

MEFs and BMDMs (biological triplicates) were mock- or JEV-infected at an MOI of 2 for 1 h. At 1 hpi, the cells were treated with DMSO or drugs for 24 h. Supernatants were then collected to measure cytokine levels of IFNβ using a CBA assay, following the manufacturer’s protocol (LEGENDPLEX MU Anti-Virus Response Panel (6-plex) Biolegend (740622). The analysis was performed using FCPA array software, and cytokine concentrations were determined based on their respective standard curves. All CBA assays were conducted at least twice, and representative data from one experiment are shown.

### IFNβ ELISA

Cell culture supernatants collected from mock- or JEV-infected BMDMs, as well as untreated or 7DHC/AY9944-treated BMDMs, were first centrifuged to remove any debris. ELISA for IFNβ was then performed according to the manufacturer’s protocol (R&D Systems, Catalog #: DY8234-05).

### Hmgcr and Dhcr7 ELISA

Mock- or JEV-infected cells, as well as those treated with poly I:C, LPS, simvastatin (10 μM), were processed for cell lysate preparation on ice for 1 h. After incubation, the lysates were centrifuged at 10,000 rpm for 10 min at 4°C. Protein concentration was determined using the BCA assay. Equal amounts of protein (30 μg) were then used for ELISA of Hmgcr and Dhcr7, following the manufacturer’s instructions (Hmgcr: MyBiosource, Cat. No. MBS7235854; Dhcr7: MyBiosource, Cat. No. MBS7606267).

### Virus entry assay through qRT-PCR

MEFs were infected with JEV at an MOI of 2 on ice for 1 h to synchronize virus binding. At 1 hpi, cells were washed and treated with 10 µM DMSO or AY9944, then incubated at 37°C for 1 h. Afterward, cells were trypsinized to remove the extracellularly bound virus. qRT-PCR was performed to assess the levels of internalized virus relative to GAPDH controls.

### Statistical analysis

Data sets were statistically analyzed using paired Student’s *t*-test. Differences were considered significant at values of *P < 0.05; **P <0.01; ***P <0.001, and ****P <0.0001. Error bars denote the mean ± SD (*n* = 3).

## Data Availability

The data are available at the Indian Metabolome Data Archive (IMDA) of the Indian Biological Data Centre (IBDC), https://ibdc.dbtindia.gov.in/imda/, under the IBDC Project accession number IMP_100037 and Study accession number IMS_100032.

## SUPPLEMENTAL MATERIAL

Data Set S1 jvi.01972-25-s0001.xlsx Peak area abundance of identified lipid species and dysregulated lipid species.

Supplemental material jvi.01972-25-s0002.pdf Figures S1 to S6; Tables S1 and S2.

ASM does not own the copyrights to Supplemental Material that may be linked to, or accessed through, an article. The authors have granted ASM a non-exclusive, world-wide license to publish the Supplemental Material files. Please contact the corresponding author directly for reuse.
