# Supplementary material for: Downmodulation of cholesterol biosynthetic network governs activation of the innate immune response to Japanese encephalitis virus infection
Source: J Virol. 2026 Feb 4;100(3):e01972-25. doi: 10.1128/jvi.01972-25 (PMC13011394; doi:10.1128/jvi.01972-25)
Supplement: Supplemental material — Figures S1 to S6; Tables S1 and S2. [file jvi.01972-25-s0002.pdf]

Supplementary Data:

Supplementary figures: - S1 to S6

Supplementary tables: - S1 to S2

A Cholesterol MS-MS

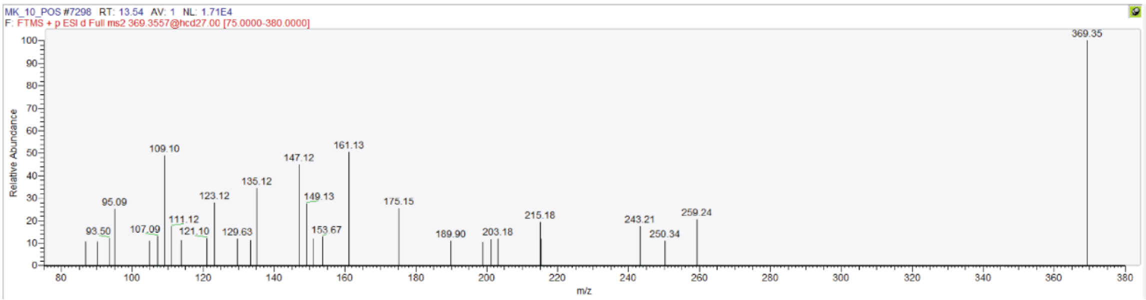

B DG MS-MS

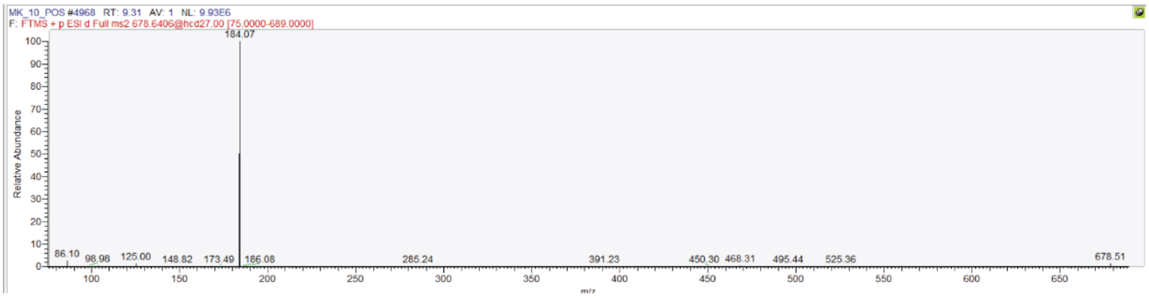

C TG MS-MS

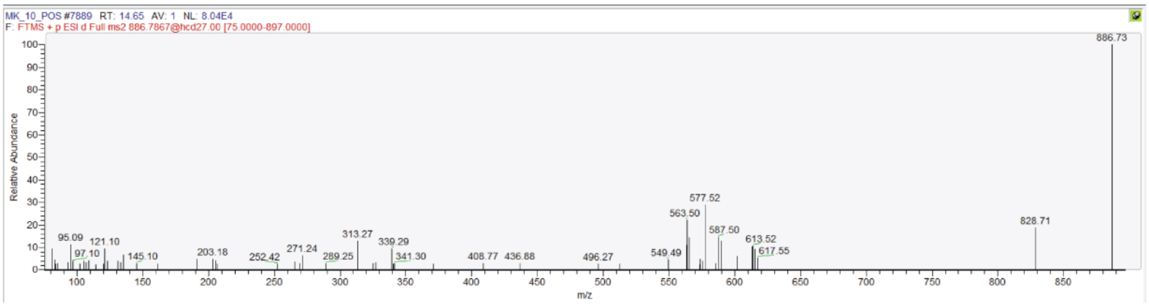

Fig. S1: MS-MS spectra overview

MS-MS spectra representing cholesterol (A), DG (B) and TG (C).

A

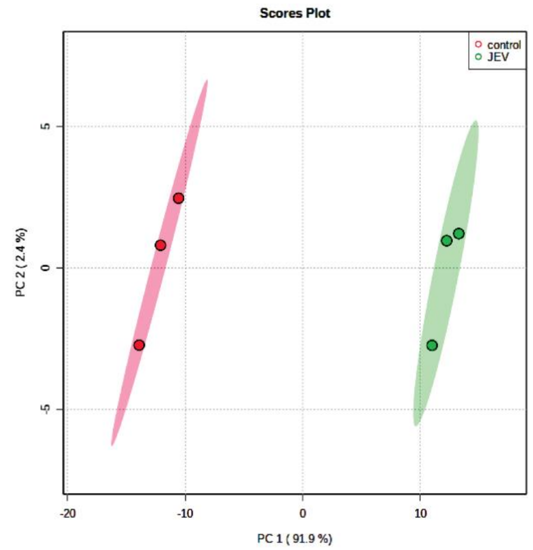

B

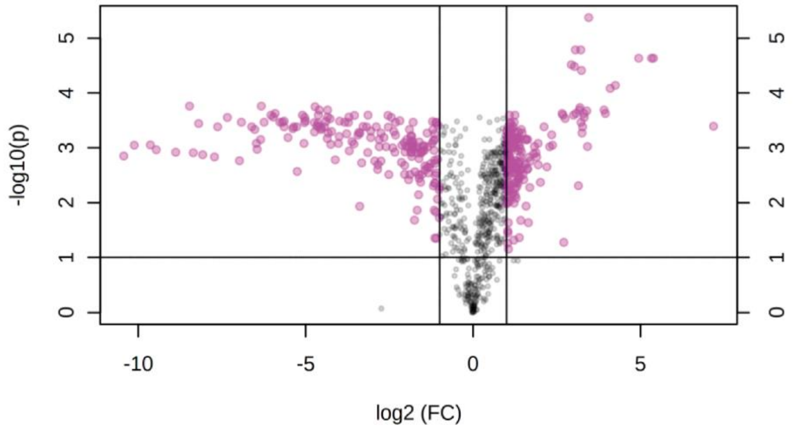

Fig. S2: Lipidome study overview

MEFs were infected with JEV at an MOI of 2 for 24 h. Each condition (UI and JEV infected MEFs) had n=3 replicates. (A) Principal Component analysis (PCA) of the total lipidome of UI and JEV infected samples. Data was normalized by sum; log transformed and mean centered. PC1 explained 91.9% of the variation, and PC2 explained 2.4% of the variation. (B) Volcano plot representing the $\log_2$ (FC) in the abundance of identified lipid species in JEV-infected cells relative to UI (JEV/UI). Pink coloured bubbles represent the lipid species with ≥ -1 and ≥ 1 $\log_2$ (FC) and p<0.05.

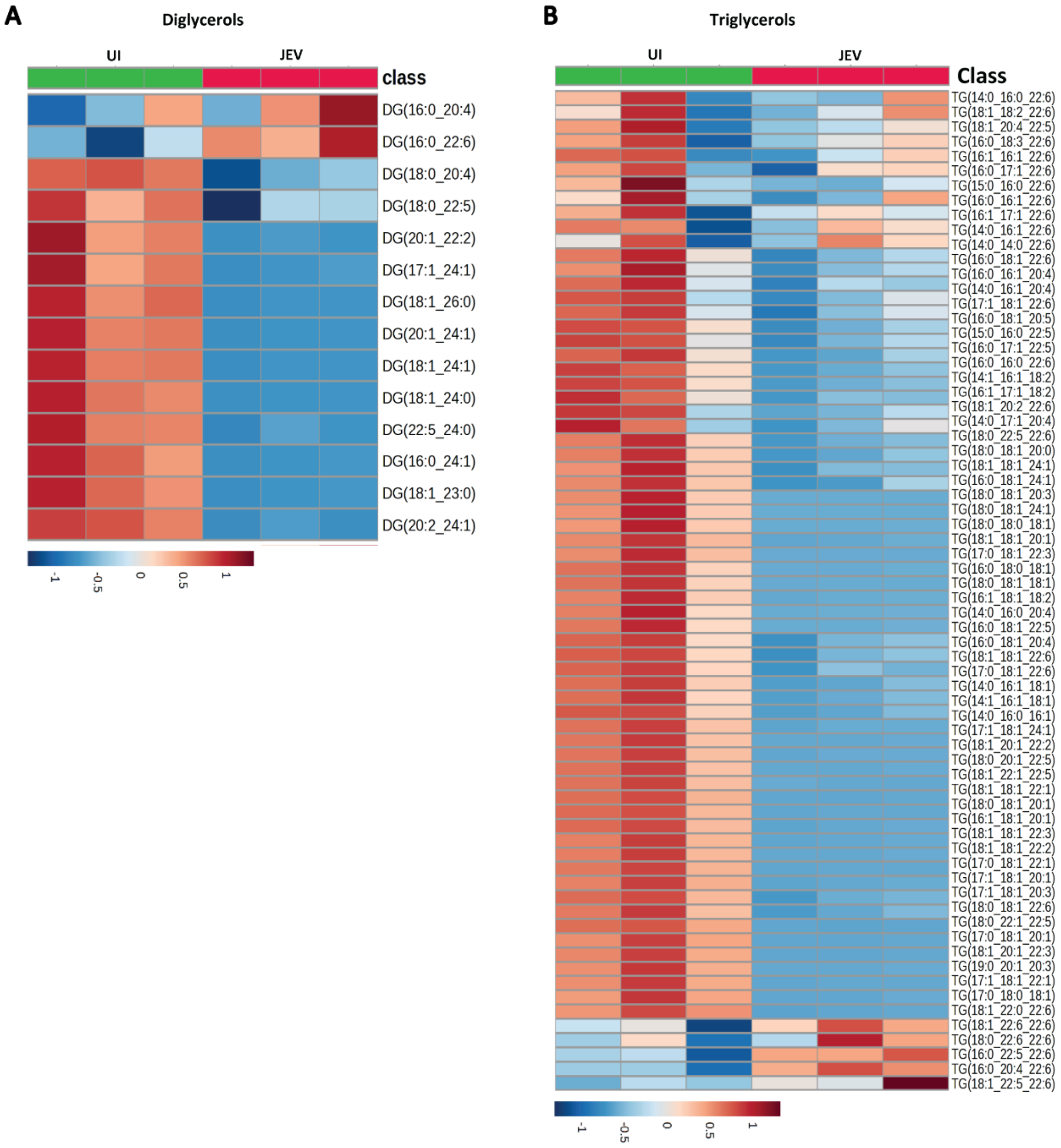

Figure S3: JEV infection downregulates glycerolipids level in MEFs

Heat map represents the abundance of diglycerol [DG] (A) and triglycerol [TG] (B) species in UI and JEV-infected MEFs. Heat map was generated using MetaboAnalyst software.

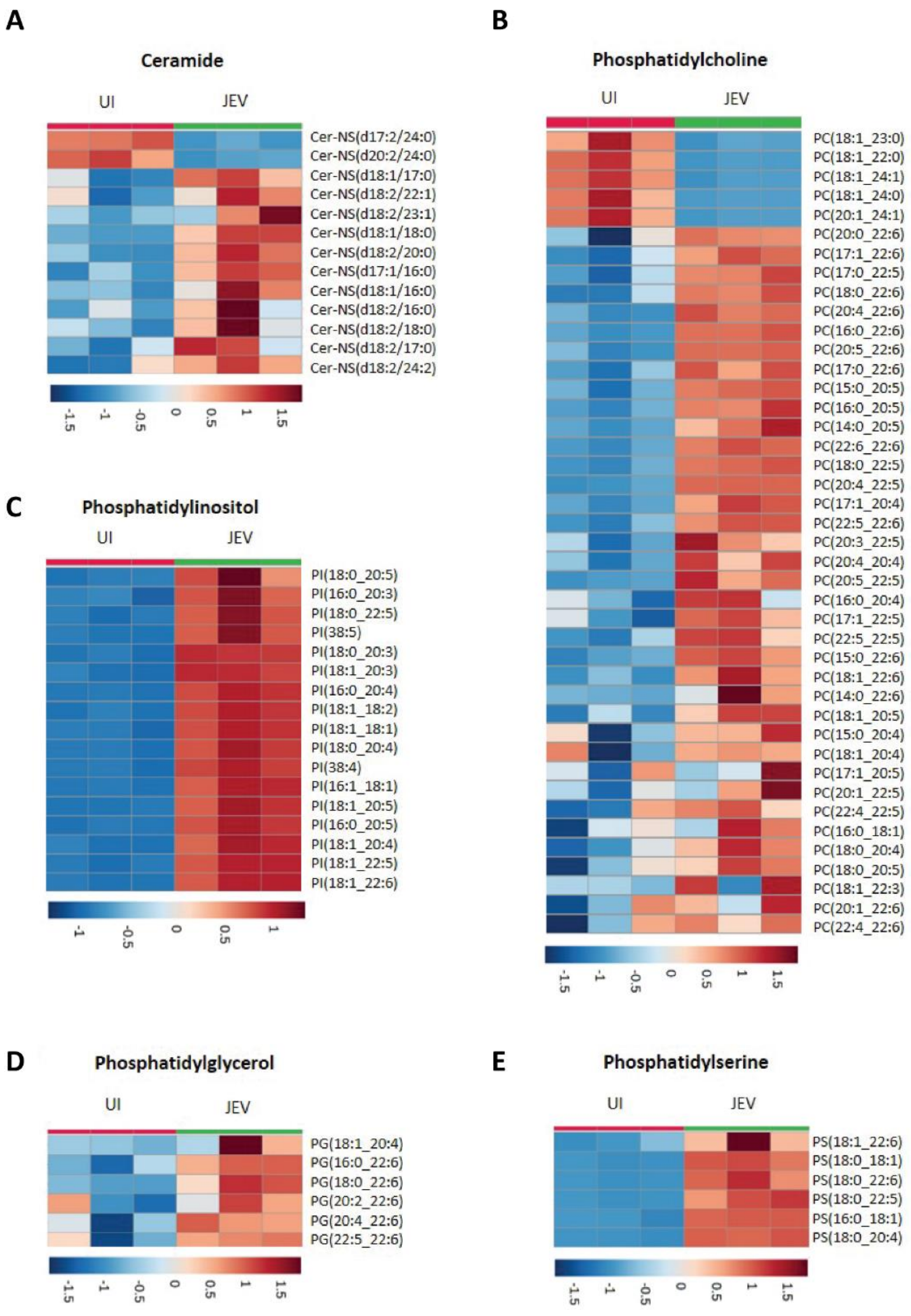

Fig. S4: JEV infection upregulates a diverse range of host ceramide and phospholipid species in MEFs

Heat map representing the abundance of ceramide (A), phosphatidylcholine [PC] (B), phosphatidylinositol [PI] (C), phosphatidylglycerol [PG] (D) and phosphatidylserine [PS] (E) lipid species in UI and JEV infected samples. Heat map analysis was done using MetaboAnalyst software.

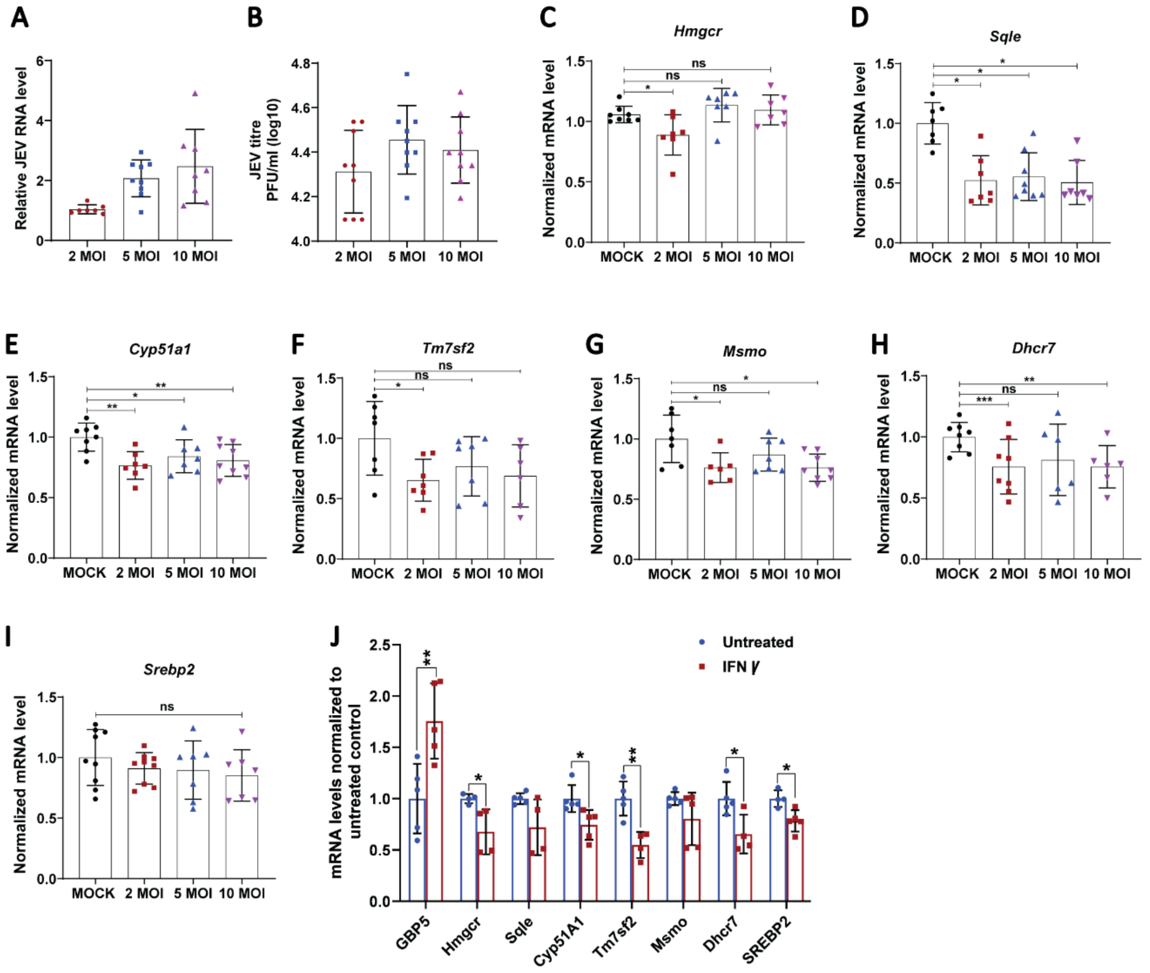

Fig. S5: JEV infection downregulates genes of the cholesterol biosynthetic pathway in BMDMs

BMDMs were MOCK/JEV infected at MOIs of 2, 5, and 10. At 24 hpi, the RNA levels of JEV (A) and cholesterol biosynthetic genes - *Hmgcr* (C), *Sqle* (D), *Cyp51A1* (E), *Tm7sf2* (F), *Msmo* (G), *Dhcr7* (H), and *Srebp2* (I) were measured using qRT-PCR, normalized to mock infected cells. Extracellular viral titers were quantified using a plaque assay (B). (J) BMDMs were treated with IFNγ (30 ng/ml) for 12 h, and mRNA levels of cholesterol biosynthetic genes were then analysed via qRT-PCR. The bar graph represents the relative gene expression levels normalized to untreated controls. The data presented is the mean ± SD of values obtained from three independent experiments. Student t-test was used to calculate P values (*P < 0.05; **P < 0.01, ****P<0.0001).

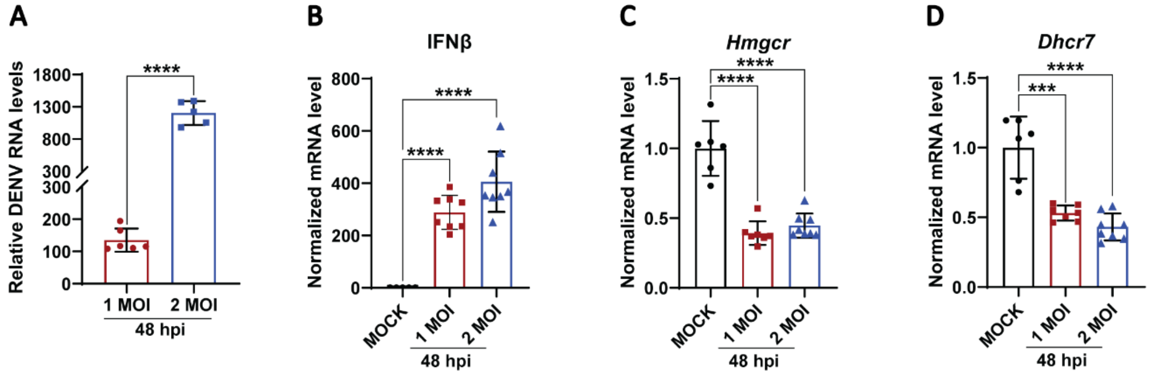

Fig. S6: Cholesterol biosynthetic pathway is transcriptionally down-modulated upon DENV-2 infection

(A-D) MEFs were infected with DENV at MOIs of 1 and 2 for 2 h. At 48 hpi, DENV RNA (A), IFNβ (B), *Hmgcr* (C) and *Dhcr7* (D) mRNA levels were measured by qRT-PCR and normalized to uninfected controls. Student t-test was used to calculate P values (*P < 0.05; **P < 0.01, ****P<0.0001).

Table S1: List of antibodies/reagents/drugs and their source

REAGENT OR RESOURCE	SOURCE	IDENTIFIER
**Antibodies**		
Alexa Fluor 568 goat anti-mouse IgG (H+L)	Invitrogen	(A-10037, dilution 1:500)
β-Actin	CST	(4970S, dilution 1:1000)
Dhcr7	Genetex	(GTX130695, dilution 1:2000)
GAPDH	Genetex	(GTX100118, dilution 1:10000)
IRF3 (D83B9)	CST	(4302S, dilution 1:1000)
JEV-NS1	Abcam	Cat #: ab41651
JEV-NS3	GeneTex	(GTX125868, dilution 1:5000)
JEV-NS5	GeneTex	(GTX131359, dilution 1:3000)
pIRF3 (S396)	CST	(29047S, dilution 1:1000)
SREBP2	Invitrogen	(PA1-338, dilution 1:1000)
**Media/Reagents/Chemicals**		
DMEM	Gibco	Cat #: 12100-046
MEM	Gibco	Cat #: 61100-061
RPMI-1640	Gibco	Cat #: 31800-022
FBS	Gibco	Cat #: 10270-106
PSG	Himedia	Cat #: A007
Trypsin EDTA solution 1 X	Himedia	Cat #: TCL007
Trizol reagent	Takara	Cat #:9109
Random hexamer	Sigma-Aldrich	Cat #:H0268
Recombinant Ribonuclease inhibitor	Promega	Cat #: N2515
ImPromIITM reverse Transcriptase	Promega	Cat #: A3803
BODIPY 493/503	Thermo	Cat #: D3922
ProLong Gold anti-fade reagent with DAPI	Invitrogen	Cat #: P36935
Sodium pyruvate	Himedia	Cat #: (TCL015)
2-Mercaptoethanol	Sigma	Cat #: (M6250-250ML)
Agarose Type VII	Sigma	Cat #: (A4018)
BCA kit	Pierce	Cat #: 23225

Dimethyl sulfoxide	Sigma	Cat #: (276855-250ML)
DL-Dithiothreitol (DTT)	SRL	Cat #: (3483-12-3)
EDTA	Sigma	Cat #: (E9884-500G)
HBSS	Gibco	Cat #: (14175095)
HEPES sodium salt	Sigma	Cat #: (H3784-500G)
Premix Ex Taq™ (Probe qPCR)	Takara	Cat #: (RR390A)
Protease inhibitor cocktail (PI)	Sigma	Cat #: (P8340)
PVDF membrane	Merck Millipore	Cat #: (IPVH00010)
RBC lysis buffer	GCC Biotech	Cat #: (19114B1076)
SDS	Sigma	Cat #: (L3771-500G)
SYBR® Premix Ex Taq™	Takara	Cat #: (RR420A)
Triton™ X-100	Sigma	Cat #: (T9284-500ML)
Tween 20	G-Biosciences	Cat #: (RC1227)
LipofectamineTM RNAimax	Invitrogen	Cat #: (13778030)
LEGENDPLEX MU Anti-Virus Response Panel (6-plex)	Biolegend	Cat #: (740622)
Mouse Dhcr7 ELISA kit	MyBiosource	MBS7235854
Mouse Hmgcr ELISA kit	MyBiosource	MBS7606267
Mouse IFN-β ELISA kit	R and D systems	Cat #: (DY8234-05)
Deoxyribonuclease I (DNase I)	SRL	Cat #: (61824)
Phenylmethylsulfonyl fluoride (PMSF)	Sigma	Cat #: (329-98-6)
IGEPAL	Sigma	Cat#: I8896
MTT 3-(4,5-Dimethylthiazol-2-yl)-2,5Diphenyltetrazolium Bromide	VWR life science	Cat#: (0793-1G)
**Drugs**		
AY9944	MedChem Express	Cat #: HY-107420
Cholesterol	Sigma	Cat #: C4951
7-dehydrocholesterol	Sigma	Cat #: 30800
Mevalonate	Cayman	Cat #: 37476
Simvastatin	Cayman	Cat# 10010344
Tamoxifen	Cayman	Cat #: 13258

Table S2: Primers used in the study.

S.No.	Gene name	Forward (5'-3') sequence	Reverse (5'-3') sequence
1.	Cyp51A1	GACAGGAGGCAACTTGCTTTC	GTGGACTTTTCGCTCCAGC
2.	DENV2	GAGAGACCAGAGATCCTGCTGTCT	ACCATTCCATTTTCTGGCGTT
3.	Dhcr7	AGGCTGGATCTCAAGGACAAT	GCCAGACTAGCATGGCCTG
4.	GAPDH	CGTCCCGTAGACAAAATGGT	TTGATGGCAACAATCTCCAC
5.	Gbp5	CAGACCTATTTGAACGCCAAAGA	TGCCTTGATTCTATCAGCCTCT
6.	Hmgcr	AGCTTGCCCGAATTGTATGTG	TCTGTTGTGAACCATGTGACTTC
7.	. IFN-α	ATGGCTAGRCTC TGTGCTTTCCT	AGGGCTCTCCAGAYTTCTGCTCTG
8.	IFN-β	AAGAGTTACACTGCCTTTGCCATC	CACTGTCTGCTGGTGGAGTTCATC
9.	JEV	AGAGCACCAAGGGAATGAAATAGT Taqman probe: CCACGCCACTCGACCCATAGACTG (5′ end FAM, 3′end TAMRA).	AATAAGTTGTAGTTGGGCACTCTG
10.	Msmo	AAACAAAAGTGTTGGCGTGTTC	AAGCATTCTTAAAGGGCTCCTG
11.	Neg qPCR	GGCCGTCATGGTGGCGAATAA	GTCAATGAGTGTTCCAAGTTCTCG
12.	NVnegVneg	GGCCGTCATGGTGGCGAATAAGAGC TTGTTGGACGGTAGAG	Underlined sequence represents non-viral tag sequence.
13.	Sqle	ATAAGAAATGCGGGGATGTCAC	ATATCCGAGAAGGCAGCGAAC
14.	Srebp2	GCAGCAACGGGACCATTCT	CCCCATGACTAAGTCCTTCAACT
15.	Tm7sf2	GTCGCGGCTTTACTGATCCT	CAGGCAGATAGGCCGGTAG
